# A new nonlinear viscoelastic model and mathematical solution of solids for improving prediction accuracy

**DOI:** 10.1038/s41598-020-58240-y

**Published:** 2020-02-10

**Authors:** Qinwu Xu, Björn Engquist, Mansour Solaimanian, Kezhen Yan

**Affiliations:** 1 School of Civil Engineering, Yango University, Fuzhou, 350015 China; 2 Oden Institute for Computational Engineering and Sciences, the University of Texas at Austin, Austin, TX 78712 USA; 3 Department of Mathematics, the University of Texas at Austin, Austin, TX 78712 USA; 4 Civil and Environmental Engineering, Penn State, State College, PA, 16801 USA; 5 School of Civil Engineering, Hunan University, Changsha, 410082 China

**Keywords:** Engineering, Mechanical properties

## Abstract

We developed an innovative material nonlinear viscoelastic model with physical mechanism and mathematical solution to improve existing ones. The relaxation modulus transits from the glassy stage to the rubbery stage through a time-dependent viscosity in a continuous spectrum considering the nonlinear strain hardening. Experimental results of differential solid materials including asphalt concrete, agarose gel, vaginal tissue, polymer, agar, bone, spider silk, and hydrogel demonstrate that the developed model is superior to generalized Maxwell model or Prony series for more accurate prediction outside of the range for data fitting while using much less model parameters. Numerical simulation results indicate that the new model has improved accuracy. It is stable numerically, and does not reduce computation speed. Therefore, the model may be used to simulate a broad range of viscoelastic solids for predicting experimental data and responses with improved accuracy.

## Introduction

**Figure 1:**
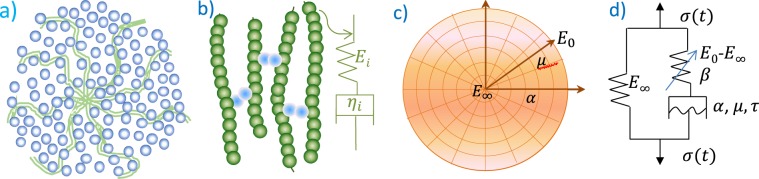
(**a**) arm-retraction model^24^; (**b**) Prony series model; (**c**) our new model, and (**d**) nonlinear spring-dashpot system.

The deformation of viscoelastic material is temperature dependent with thermal transition. Free volume changes or relaxation time are used to describe this transition behavior. Viscoelastic deformation has been interpreted by two atomistic mechanisms^1^: (1) distortion of chemical bonds’ lengths and angles connecting atoms in a small and quick motion; (2) large-scale rearrangements of atoms and molecules. The thermal transitions with elevated temperatures may involve a few stages (e.g. for polymers) including the $\gamma$ (local motion of molecular), $\beta$(bend/stretch of molecular), glass (from the glassy to the rubbery stage), and terminal transitions (melts into liquids)^2^. Different materials involve different transitions, but glass transition is the majority format of deformation for viscoelasticity as the focus of this study. Material viscoelasticity can be characterized by relaxation modulus $E(t)$ or dynamic modulus ${E}^{\ast }(\omega )=E{\prime} (\omega )+iE{\prime\prime} (\omega )$. $E(t)$ and ${E}^{\ast }(\omega )$ are dependent on temperature which can be converted to an equivalent time or frequency according to the time-temperature superposition principal. Researchers have developed physical models to describe the (linear) viscoelastic behavior. Based on the molecular dynamics theory, the Rouse model^3^ uses the Brownian motion theory to simulate the single chain diffusion of beads that are connected via harmonic springs. The Kremer-Grest model^4^ uses up to hundreds of chains^5^ for simulating polymer elements. The single-chain theories such as the tube theory^6^ and arm retraction model with arm-starts^7^ (see Fig. 1a) have also been proposed to describe the linear viscoelastic behavior of entangled polymers. Among these models, the generalized Maxwell (GM) model appears to be mostly used for describing the glass transition of linear viscoelastic solids. For the unentangled polymers the spring-dashpot series of GM model physically represent different molecular chains with variable lengths under time distributions^1^ (see Fig. 1b). $E(t)$ of GM model is expressed as:

1 $$E(t)={E}_{\infty }+\mathop{\sum }\limits_{i=1}^{n}{E}_{i}{e}^{-\frac{{E}_{i}}{{\eta }_{i}}t}$$

where ${E}_{\infty }$ is the modulus at infinite time, ${E}_{i}$ is the elastic modulus of the spring, ${\eta }_{i}$ is viscosity of the linear dashpot in series, and $n$ is the number of spring-dashpot terms. This summarized exponential terms in a discrete spectrum is also named as Prony series (PS). It becomes a standard solid model when $n=1$. The Maxwell or GM-based model and its PS formula have been widely adopted to fit modulus of linear viscoelastic materials^8^. These materials include polymers^9^,dielectric elastomers^10,11^, glasses^12^, silicon^13^, tissue^14^, brain^15^, ligament^16^, blood vessels^17^, worm^18^, and asphalt concrete (AC)^19^. GM model has also been used as a base to be extended to plasticity model including the viscoplastic Bingham–Maxwell model^20^. Other formulas similar to GM model includes the (generalized) Kelvin model and Burgers model^21^. PS is computationally efficient with its exponential formula for time integration^22^, and thus it has been implemented as a standard material model in most numerical software such as the ANSYS and ABAQUS for simulating material and structural responses. The nonlinear viscoelastic models have also been proposed for simulating materials with large deformation or properties that change with deformation or time^23,24^. For example, Schapery’s model has considered the spring’s elastic modulus as a nonlinear function of time^25^, and some other models have presented the dashpot’s stress in a nonlinear function dependent on the strain rate^26,27^ or relaxation time^28^. Please note that our literature review doesn’t cover fluid models.

However, a few critical questions arise. As discussed above, the models based on the theory of multiple molecular chains were derived and validated primarily for polymers, and they are less suitable for describing the physics of other materials with different morphology (e.g. asphalt with amorphous structure). For the GM model or PS: (1) the PS formula can produce an instability in fitting experimental data^19,29^ as will be discussed later; (2) it is relatively difficult to determine a great number of model parameters using experimental data, (3) with a great number of model parameters the accuracy for fitting experimental data has improved mathematically. However, the physical interpretation of this large spring and dashpot system becomes less clear and more challenging.

These shortcomings of existing models are our motivations to seek development of a new material viscoelastic model that represents a broad range of materials more accurately. We have developed a theoretical model and mathematical solution. We conducted experimental validations for a number of materials that range from the inorganics to biomaterials. We have demonstrated that the model has improved the accuracy in both fitting experimental data as well as predicting modulus outside of the experimental range. The model is stable numerically, and it doesn’t reduce computation speed. It uses much less model parameters than the GM model or PS. We also account for the nonlinear strain-hardening behavior in the proposed model.

## Results and Analysis

### Proposed new material model

Micro-mechanisms and network-based models have been proposed for simulating material viscoelasticity in polymer science. For example, the coarse-grain-polymer network model represents the stretched mesh-like networks in a viscosity medium^30^, and the micro-network model symmetrically grows like tree^31^. We proposed a network model in a relatively simple format to interpret the physical mechanism of general materials as shown in Fig. 1c. The model domain consists of an elastic network and viscous media or “fluid” filled within the network branches. The elastic network carries a modulus ranging from the minimum ${E}_{\infty }$ ($t=\infty )$ at rubbery stage to the maximum ${E}_{0}$ ($t=0$) at glass stage.

Viscosity may be dependent on time and strain rate. E.g., it may follow a power-law function dependent on strain rate^32^ or a linear function of time^33,34^. Here we proposed the viscosity model following a power function of time to consider its nonlinear behavior, as follows:

2 $$\eta (t)=\mu {[\frac{(t-{t}_{0})}{\tau }]}^{\alpha }$$

where *t*_0_ is a reference time (s), $\,\alpha$ is model parameter, $\mu$ is a viscosity parameter (MPa.s), and $\tau$ (s) is a retardation time (s) that accounts for temperature change as well as vanishes the unit of absolute time $(t-{t}_{0}$). $\tau =1\,$s when ${T}={{T}}_{0}$ as constant, where ${T}_{0}$ is the reference or constant temperature. When $T\ne {T}_{0}$ $\tau \,$can be estimated using the WLF rule for time-temperature superposition as $log\tau =-\frac{{C}_{1}(T-{T}_{0})}{[{C}_{2}+(T-{T}_{0})]}$. Energy dissipation is produced by internal friction according to the interfacial friction theory-based viscous mechanism^35^. Viscosity is essentially a “fluid” friction^35^ that transforms kinetic energy to heat through the interfacial friction. Therefore, we may understand $\mu$ as a friction coefficient between the elastic network and viscous media in this model. A higher $\mu$ value induces a higher force of friction, resulting in relatively higher $E\,$value (Supplementary Fig. 1a). The fractional number ${\rm{\alpha }}$ indicates the time or thermal sensitivity (here “thermal” has the equivalent effect of relaxation time according to the time- temperature superposition rule. A higher $\alpha$ value indicates a higher modulus variation rate $\partial E(t)/\partial t$ (Supplementary Fig. 1b). The fractional number and derivatives are related to molecular theories describing the macroscopic behavior of viscoelastic materials^36^.

The model can also be interpreted mathematically by a nonlinear spring (elastic network) and dashpot (viscosity) system as shown in Fig. 1d.

Applying a constant strain ${\varepsilon }_{0}H(t)$ on the model shown in Fig. 1d ($H(t)$ is the Heaviside step function that $H(t)=1$ when $t\ge {t}_{0}$ and $H(t)=0$ when $t < {t}_{0}$), the stress equilibrium satisfies the followings:

3 $${\varepsilon }_{0}H(t)={\varepsilon }_{1}+{\varepsilon }_{2}$$

4 $$\sigma (t)={E}_{\infty }{\varepsilon }_{0}H(t)+{E}_{R}{\varepsilon }_{1}$$

5 $${E}_{R}{\varepsilon }_{1}=\eta (t){\dot{\varepsilon }}_{2}(t)={\rm{\mu }}{[(t-{t}_{0})/\tau ]}^{\alpha }{\dot{\varepsilon }}_{2}(t)$$

where ${\varepsilon }_{1}$ and ${\varepsilon }_{2}$ are strain of the spring and dashpot, respectively, and ${E}_{R}={E}_{0}-{E}_{\infty }$ is the modulus variation range.

Substitute Eq. (3) into (5), ${\varepsilon }_{1}$ can be solved as follows:

6 $${\varepsilon }_{1}={\varepsilon }_{0}H(t){e}^{-\frac{\tau {E}_{R}}{(1-\alpha ){\rm{\mu }}}{[\frac{(t-{t}_{0})}{\tau }]}^{1-\alpha }}$$

Substitute Eq. (6) into Eq. (4), $E(t)$ can be derived as follows:

7 $$E(t)=\sigma (t)/{\varepsilon }_{0}H(t)={E}_{\infty }+{E}_{R}{e}^{-\frac{\tau {E}_{R}}{(1-\alpha ){\rm{\mu }}}{[\frac{(t-{t}_{0})}{\tau }]}^{1-\alpha }}$$

$E(t)$ starts at the plateau modulus ${E}_{0}$ at the glassy stage ($t={t}_{0}$), relaxes with time via the viscosity reaction rate and time sensitivity parameter $\alpha$, and then transits to ${E}_{\infty }$ at the rubbery stage until the infinite time.

At the frequency (ω) domain, the viscosity can be expressed as follows:

8 $${\rm{\eta }}({\rm{\omega }})=\frac{\mu }{\tau }{(i\omega \tau )}^{1-\alpha }$$

Substitute $i={{\rm{e}}}^{+{\rm{i}}\frac{{\rm{\pi }}}{2}}\,$into Eq. (8), under the sinusoidal loading signal the stress-strain relationship can be expressed as:

9 $${\rm{\sigma }}\{{e}^{i\omega }\}={{\rm{e}}}^{+{\rm{i}}\phi }\frac{\mu }{\tau }{({\rm{\omega }}{\rm{\tau }})}^{1-{\rm{\alpha }}}\varepsilon \{{e}^{i\omega }\}$$

where $\phi =(1-\alpha )\pi /2\in (0,\frac{\pi }{2})$. According to the stress equilibrium the complex modulus ${E}^{\ast }(\omega )$ of the model can be derived as follows:

10 $${E}^{\ast }(\omega )={E}_{\infty }+\frac{{E}_{R}\mu /\tau {{\rm{e}}}^{i\phi }{(\omega \tau )}^{1-\alpha }}{{E}_{R}+\mu /\tau {{\rm{e}}}^{i\phi }{(\omega \tau )}^{1-\alpha }}$$

Decompose ${{\rm{e}}}^{{\rm{i}}\phi }=cos\phi +{\rm{i}}sin\phi$, ${E}^{\ast }(\omega )$ can be re-derived as follows (Supplementary information: Model derivation for complex modulus):

11 $${E}^{\ast }(\omega )={E}_{\infty }+\frac{{E}_{R}\mu /\tau {(\omega \tau )}^{1-\alpha }[\cos (\phi )+\,\sin (\phi )i]}{{E}_{R}+\mu /\tau {(\omega \tau )}^{1-\alpha }[\cos (\phi )+\,\sin (\phi )i]}$$

In comparison, the complex modulus of GM model is expressed as^19^:

12 $${E}^{\ast }(\omega )={E}_{\infty }+\mathop{\sum }\limits_{1}^{n}\frac{{\omega }^{2}{E}_{i}{\eta }_{i}^{2}+i\omega {E}_{i}^{2}{\eta }_{i}}{{E}_{i}^{2}+{\omega }^{2}{\eta }_{i}^{2}}$$

The model becomes a standard solid model ($n=1$) when $\alpha =0$ and $\phi =\pi /2$. To consider more parallel series like PS does, our model can also be extended as follows:

13 $$E(t)={E}_{\infty }+\sum _{{\rm{i}}}{E}_{i}{e}^{-\frac{{E}_{i}{\tau }_{i}}{(1-{\alpha }_{i}){{\rm{\mu }}}_{{\rm{i}}}}{[\frac{(t-{t}_{0})}{{\tau }_{i}}]}^{1-{\alpha }_{i}}}$$

where ${E}_{i}$ is the modulus of the ${i}^{th}$ parallel series of the system. The extended model may not be necessarily warranted for improving the accuracy of fitting experimental data, but it may offer a more flexible structure to fit and predict modulus in a wide range of relaxation time as if needed. We didn’t derive the model function from experimental data, instead the model parameters can be determined by fitting the model formula on the measured experimental data of modulus. This approach is similar to some other models including the popular Prony series, for which the model parameters including viscosities are not directly measured instead back-calculated by fitting on the measured experimental data of $E(t)$ or ${E}^{\ast }(\omega )$.

Strain hardening may occur for viscoelastic material. The model can be extended to consider the nonlinear strain hardening behavior of the elastic network ${E}_{R}$ as follows:

14 $$E(t)=\sigma (t)/{\varepsilon }_{0}H(t)={E}_{\infty }+{(1+\varepsilon )}^{\beta }{E}_{R}{e}^{-\frac{\tau {E}_{R}}{(1-\alpha ){\rm{\mu }}}{[\frac{(t-{t}_{0})}{\tau }]}^{1-\alpha }}$$

$\beta$ is a coefficient to account for the nonlinear strain hardening. All model parameters are determined by fitting on experimental data using optimization method.

The proposed model may satisfy the thermodynamic consistency (Supplementary information: Thermodynamic consistency) to ensure nonnegative energy dissipation. The viscosity is more often dominated by shear modulus^37,38^, and thus the shear and bulk relaxation modulus $G(t)$ and $K(t)$ are often not equivalent. However, in our numerical implementation we considered Poisson’s ratio $v\,$as constant which is the often case for engineering analysis. Therefore, $G(t)$ and $K(t)$ can be derived following the same formula as $E(t)$ according to their linear relationship^1,28^^,^ $G=E/2(1+v)$ and $K=E/3(1-2v)$.

### Numerical solution method

Consequently, we developed a numerical solution to implement the proposed model for simulating responses of materials and structures. Numerical solution methods for the PS were presented in existing literature. To implement the model in numerical solution, different methods exist with their own advantages or disadvantages. Either the time domain or frequency domain can be used in the numerical solution^39,40^. Our objective for the numerical implementation is to obtain similar computation speed and numerical stability versus the PS as the basis. We developed a Galerkin-based finite element (FE) scheme within a time domain, which is robust and fast for computation without requiring transformation as the frequency-domain method does.

For a solid under a dynamic loading, we can express the stress equilibrium based on a strong form as follows:

15 $$\nabla \cdot {\boldsymbol{\sigma }}(t)+b=\rho \frac{{\partial }^{2}u(t)}{\partial {t}^{2}}on\,\Omega $$

where ${\boldsymbol{\sigma }}(t)$ is a viscoelastic stress tensor at time $t\in \,[0,\,{t}_{d}]$ that ${t}_{d}$ is the end of loading time, $\rho$ is the density, $b$ is the force of body weight, and $\Omega$ is the space domain. ${\boldsymbol{\sigma }}(t)$ can be expressed as follows^39^:

16 $${\boldsymbol{\sigma }}(t)={\int }_{{{\rm{t}}}_{0}}^{{\rm{t}}}R(t-\tau )\dot{\varepsilon }(\tau )d\tau $$

where $R$ is relaxation modulus matrix as a function of $E(t)$, $G(t)$ and $K(t)$. Following the Galerkin method, multiply a test function $p(t)$ and then apply space integrations on Eq. (15):

17 $${\int }_{{\Omega }}(\nabla \cdot {\boldsymbol{\sigma }}(t))\cdot {\boldsymbol{p}}(t)d{\Omega }+{\int }_{{\Omega }}b\cdot {\boldsymbol{p}}(t)d{\Omega }={\int }_{{\Omega }}\rho \ddot{u}(t)d{\Omega }$$

Substitute Eqs. (16) to (17) and apply Green’s function, the following weak form can be attained:

18 $${\int }_{{\Omega }}[{\int }_{{t}_{0}}^{t}R(t-\tau )\dot{u}(\tau )d\tau -\rho \ddot{u}(t)]d\Omega =\Re (t)$$

where $\Re (t)={\int }_{\partial \Omega }f(t)ds-{\int }_{\Omega }bd\varOmega$ is the loading vector, and $f(t)$ is the external loading applied on the surafce $\partial \Omega$ as a natural bounary conditon.

For numerical solution purpose the time integration and differential are discretized to finite time steps. Different methods exist for nuermical approximation of differentials, and here we used the Eurler’s rule and backward method to discretize Eq. (18) as follows:

19 $$\mathop{\sum }\limits_{j=1}^{n}{\int }_{\Omega }{\int }_{{{\rm{t}}}_{{\rm{j}}-1}}^{{t}_{j}}R(t-\tau )d\tau [u(j)-u(j-1)]/\Delta td\Omega -{K}_{m}[u(n)+u(n-2)-2u(n-1)]=\Re (t)$$

where ${K}_{m}={\int }_{\Omega }\rho d\Omega /\Delta {t}^{2}$, and $\Delta t$ is the time step length. A viscoelastic stiffness matrix can be defined:

20 $${K}_{{\rm{ve}}}(j):={\int }_{\Omega }{\int }_{{{\rm{t}}}_{{\rm{j}}-1}}^{{t}_{j}}R(t-\tau )d\tau /\Delta t$$

Set reference time ${t}_{0}=0$, the mathematical solution of ${K}_{{\rm{ve}}}(j)$ for the proposed model at the one-dimensional domain is derived as follows:

21 $$\begin{array}{rcl}{K}_{{\rm{ve}}}(j) & = & {\int }_{{t}_{j-1}}^{{t}_{j}}[{E}_{\infty }+E{(1+\varepsilon )}^{\beta }{e}^{-\frac{({E}_{0}-{E}_{\infty })}{(1-\beta )\mu }{(t-\tau )}^{1-\alpha }}]/\Delta td\tau \\  & = & {E}_{\infty }+\frac{1}{\alpha }{(1+\varepsilon )}^{\beta }{[\frac{({E}_{0}-{E}_{\infty })}{(1-\alpha )\mu }]}^{-\frac{1}{a}}{\rm{Gamma}}[\frac{1}{\alpha },\frac{({E}_{0}-{E}_{\infty })}{(1-\alpha )\mu }{(t-\tau )}^{a}]/\Delta t\end{array}$$

where ${\rm{Gamma}}$ is the incomplete gamma function for $\tau \in [{t}_{j-1},{t}_{j}]$. Substitute 22 to Eq. (19) which can be rearranged as follows:

22 $$u(n)[{K}_{{\rm{ve}}}(n)-{K}_{m}]=\Re (t)+\mathop{\sum }\limits_{j=1}^{n}{K}_{{\rm{ve}}}(j)u(j-1)-\mathop{\sum }\limits_{j=1}^{n-1}{K}_{{\rm{ve}}}(j)u(j-1)+{K}_{m}[u(n-2)-2u(n-1)]$$

Thus $u(n)$ at the current or ${n}^{th}\,\,$time step can be solved from this system for $n=1,2,3..N$. Follwing the deformation-strain-stress constitutive relationship, the responses of strain and stress can also be solved.

### Experimental evaluation

**Figure 2:**
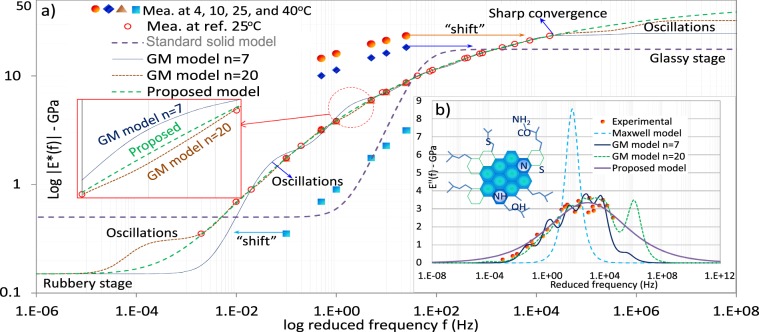
Dynamic modulus E^*^(*f*) and model fits: (**a**) $|{{E}}^{\ast }({f})|$ and (**b**) loss modulus *E*′′: the proposed model has obtained higher fitting accuracy than GM model that uses a much larger term number of *n* = 7 and 20.

We conducted experimental validations on different materials including inorganics, biomaterials and tissues. We conducted dynamic modulus tests on the AC material, at four temperatures (5, 10, 25, and 40 °C) and six frequencies (0.1, 0.5, 1, 5, 10, and 25 Hz). The measured modulus values of $|{E}^{\ast }(f)|$ at different temperatures can be converted to that at a reference temperature (25 °C) in a reduced frequency based on the WLF temperature-time/frequency superposition rule. Figure 2a plots the experimental results of $|{E}^{\ast }(f)|$ and its model fittings using the generalized reduced gradient optimization algorithm. We compared the optimization results of the proposed model and GM model or PS (see Supplementary A). Asphalt material is amorphous and has a low molecular weight (see the molecular structure example of a crude asphalt in Fig. 2b). Thus, we may not represent its structure with individual segment components or polymer-chain-like fractions^41^, and therefore its physical behaviors may not be properly interpreted by polymer models and the PS. Results have shown that the new model is able to fit experimental data accurately with a smooth curve. It is not surprising that the standard solid model (*n* = 1) can only fit a small frequency range of the experimental data. With *n* ≥ 7 (total ≥ 15 model parameters), GM model can capture almost a full frequency range of experimental data. However, it produces local “oscillations”, and at the high frequencies $|{E}^{\ast }(f)|$ converges to ${E}_{0}$ very sharply beyond the experimental range, which seems less reasonable than expected. The average norm value of the 2nd order differential $\,{\Vert \frac{{\partial }^{2}|{E}^{\ast }(f)|}{\partial {f}^{2}}\Vert }_{{L}^{2}}\,$of the GM model is significantly higher than that of the proposed model (i.e. 52,7727 *vs*. 33,7372 MP/Hz^2^). The higher the norm differential, the lower fitting accuracy is. Fig. 2b plots the loss moduli $E\text{'}\text{'}(f)$ within the frequency domain, in which again GM model produces local “oscillations” even with a fairly large number of terms ($n$ = 20). Existing literature of this subject matter does not provide a proper interpretation of this phenomenon. In comparison, the proposed model produces smooth ${E}^{\ast }(f)$ fit on experimental data. The $|{E}^{\ast }(f)|$ value also gradually and smoothly converged when transmitting from the glassy stage to the rubbery stages outside of experimental data range. This result clearly illustrates that the proposed model has improved both the fitting and prediction accuracy as compared to GM model.

**Figure 3:**
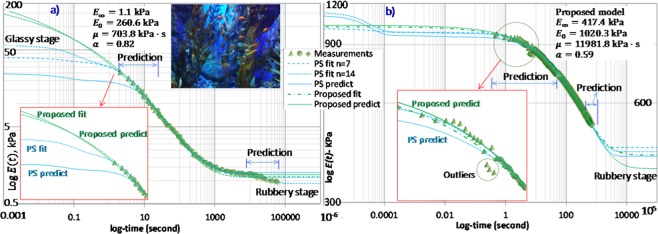
Model fits and predictions of relaxation modulus *E*(*t*) using proposed model and Prony series (PS): (**a**) agarose gel and (**b**) vaginal tissues: the proposed model improves accuracy in both experimental data fitting and *E*(*t*) predictions outside of experimental range.

We also evaluate the proposed model versus GM model for both fitting and predicting $E(t)$ of other materials including polymer, agar, bone, and tissue. Model parameters are determined by fitting on experimental data using generalized reduced gradient optimization algorithm. It is known that optimization may achieve different model parameters to satisfy the objective function. Our results have shown that the proposed model achieves more unique and stable optimization results than GM model even using the same number of model parameters, i.e. $n=2$ for GM model with five model parameters (Supplementary Fig. 3). Generally, the fitting accuracy of GM model increases with a higher term number, but it may produce more variability of output parameters without a unique solution. We used a term number of 14 (total 29 model parameters) of GM model for the following validations since it can achieve satisfied accuracy on fitting experimental data. When only part of the experimental data are used for fitting model parameters, the rest of the data is used for predictions as validation. Figure 3a presents model fitting and predictions on experimental $E(t)$ of agarose gel (data was reproduced from literature^42^, agarose is a polysaccharide polymer derived from seaweeds^43^). Figure 3b presents model fitting and prediction for vaginal tissue (data was reproduced from literature^44^). Again, the PS (corresponding to GM model in frequency domain) produces instability with many local “oscillations”, as well as sharp transitions from the glassy stage to the rubbery stage beyond the experimental range which is likely false prediction. In comparison, the proposed model achieves very smooth curve fitting on experimental data. It also more accurately predicts the modulus value which gradually converges.

Secondly, the middle time-range of experimental data is used for fitting model parameters which are then used to predict $E(t)$ at the lower and higher time ranges of experimental data. Results indicate the proposed model achieves fairly accurate predictions. In comparison, PS under-predicts $E(t)$ at low $t$ while over-predicts $E(t)$ at high $t$ as it tends to converge to ${E}_{0}$ and ${E}_{\infty }$ more sharply than expected as shown in Fig. 3. Based on the thermodynamics theory, modulus of the viscoelastic materials is expected to grow and transmit smoothly when no sudden triggering occurs to change molecular structures^45^, which conflicts with the PS prediction results. The root mean square ($RMS=\sqrt{{\sum }^{}{[({E}_{i}-{E}_{i,{\rm{predict}}})/{E}_{i}]}^{2}/N}\,$) of the proposed model is 7.0% and 0.8% for the agarose gel and vaginal tissue, respectively, which are apparently lower than that of PS (12.0% and 1.2%), indicating its improved accuracy of predictions (see Fig. 3b). By using the extended model in Eq. (13) to fit experimental data, the accuracy has very slight improvement in fitting experimental data (e.g. for the agarose gel, the ${R}^{2}$ is 4.51%, 4.34%, and 2.10%, for $n$ = 1, 2, and 3, respectively), indicating that a single series with only a few model parameters is capable of affording satisfied accuracy for both data fitting and prediction.

Validations on agar and bone materials have shown similar findings (Supplementary Figs. 4 and 5). The model parameters of PS for all these materials are presented in the supporting information (Supplementary Table 2).

### Numerical modeling results

**Figure 4:**
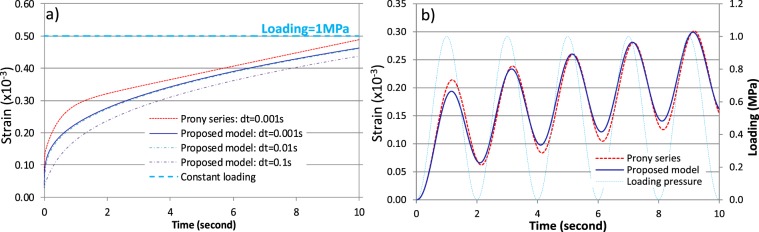
Simulated deformation: (**a**) under constant loading (*dt* = 0.01 and 0.001s) and (**b**) under sinusoidal loading (*dt* = 0.001 s): the proposed model attains different simulation results from Prony series due to its different (or more accurate) model fitting on experimental data of relaxation modulus.

After the theoretical demonstration and experimental validation, we implemented the proposed model for simulating responses of materials. We developed a computer coding for simulating responses in one-dimensional domain. We implemented the three-dimensional (3-D) model with user-defined material model in ANSYS software, in which the modulus and viscosity are defined as functions of relaxation time. We considered Poisson’s ratio as constant, and thus calculated $G(t)$ and $K(t)$ from $E(t)$ values based on their linear relationships for the 3-D model. Figure 4a,b present the simulated axial deformations of AC material under a constant loading pressure (1 MPa) and a sinusoidal loading with an amplitude of 1 MPa, respectively, for $t\in [0,10\,{\rm{s}}]$ and time step length $dt\in [0.1,\,0.001\,{\rm{s}}]$. This case study indicates that at some times the simulated deformations using the proposed model can be significantly different from that of PS. For example, a maximun deformation difference of 0.06 mm (27.4%) and 0.022 mm (12.2%) of these two models is obsered at the time of max deformation for the constant and sinusoidal loading, respectively. These differences are primarily induced by the model fit errors of the PS (e.g. local oscillatinos and sharp transitions).

For PS model, the simulation accuracy may increase with a larger number of model parameters. However, it has been usual to use a term number of $n\le 7$ for PS in numerical practice for simplicity purose^46,47^, which may cause potential numerical errors. Resutls also indicate that the developed model is always numerically stable. Simulation accuracy increaes with reducing $dt$, but longer computation time. We have tested variable $dt$ and find that the proposed model converges at $dt=0.01\,{\rm{s}}$, the same as that of PS. This indicates that the proposed model does not require longer computation time than the PS. Simulated results clearly show the time lag of deformation to loading due to viscoelastic effect (see Fig. 4b).

**Figure 5:**
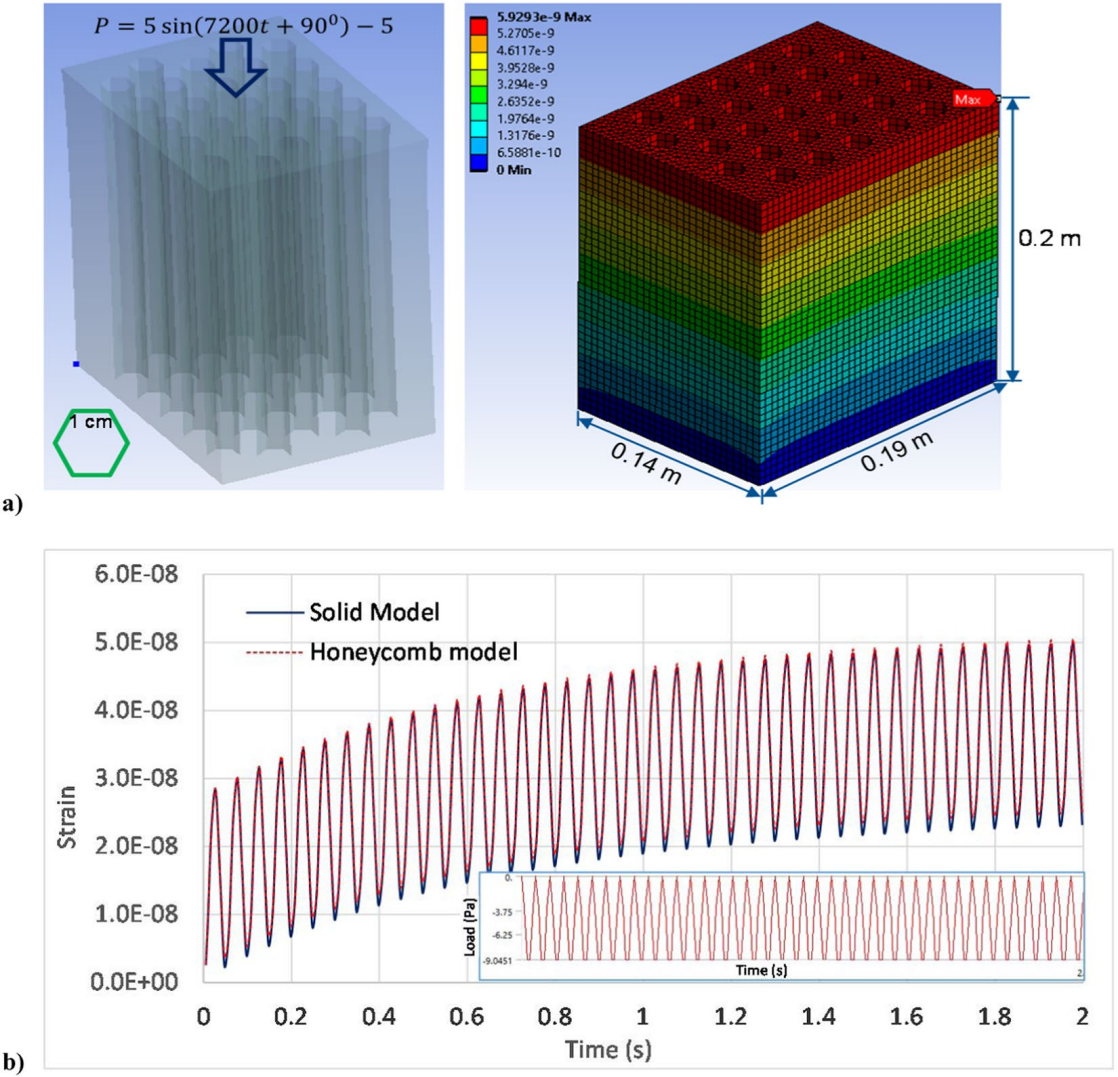
Simulation of 3-D honeycomb structure deformation under 40-cycles sinusoidal loading pulse: (**a**) hexagon honeycomb structure, FE mesh, and contour of simulated vertical deformation at the end of time, and (**b**) simulated max deformation is slightly larger than that of the solid model without honeycomb holes.

We performed a 3-D dynamic viscoelastic simulation of a hexagon honeycomb structure made of agarose gel (see Fig. 5a), with inputs of $E(t)$ exhibited in Fig. 3 and a density of 924 kg/m^3^. A sinusoidal loading pulse $P=5\,\sin (7200t+90^\circ )-5$(Pa) for $t\in [0,\,2s]\,$with 20 cycles was applied on the top. All degrees of freedom are fixed at the bottom. We also simulated a solid model without hexagon holes with the same model size for comparison. Simulated deformation increases with time and remains positive at the end of time with zero loading (see Fig. 5b), illustrating the viscoelastic behavior with energy dissipation. It is known that honeycomb structure is excellent for sustaining moment loading. Our results show that the honeycomb structure is also a good option for sustaining compression loading, as it only cause slightly larger (i.e. 1.9%, see Fig. 5b) or ignorable deformation than the full solid model without honeycombs, while it has 26.1% less weight and two-times free surface area.

**Figure 6:**
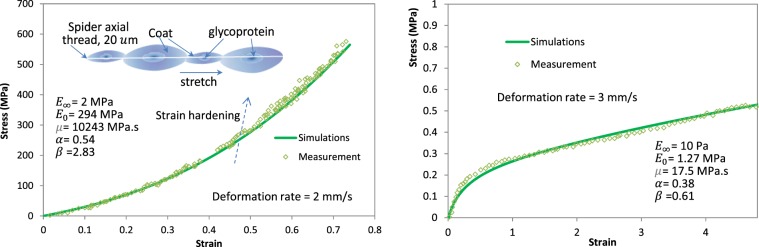
Simulated nonlinear stress-strain relationship: (**a**) simulated stress-strain behavior of spider silk showing nonlinear strain hardening behavior; (**b**) simulated nonlinear stress -strain behavior of the viscoelastic hydrogel.

We also simulated the nonlinear stress-strain relationship of a spider silk thread and a polyampholyte-formed hydrogel material, taking into account the nonlinear strain hardening. In the lab test the spider silk was subjected to a stretching loading (data reproduced from literature^48^ with permission, see Fig. 6a). The hydrogel displays viscoelastic behavior with stress recovery even at very large strains (laboratory data reproduced from literature^49^ with permission). Results have shown that the proposed model is able to capture the nonlinear strain hardening behavior, and can accurately simulate the stress-strain curves, as illustrated in Fig. 6. It shall be noted that these materials may perform plastic deformations and fractures at larger strain ranges, which are not studied in this paper.

## Discussion

We developed an innovative material nonlinear viscoelastic model to describe glass transition of solid materials for overcoming shortcomings of existing models. The model describes modulus with only five to six (w/o one additional parameter to consider nonlinear strain hardening) model parameters in a continuous spectrum. Experimental validations on different material types have demonstrated that the new model has improved accuracy in both fitting experimental data as well as predicting relaxation modulus beyond the experimental range as compared to GM model or PS which is the widely-used model for solid materials. Accurate prediction of modulus could be very useful since laboratory tests can only capture a limited range of reduced frequency or time.

The new model is able to predict $E(t)$ outside of the experimental range more accurately with a smoother curve than the PS. The predicted curve captures the glass transition more naturally and smoothly than the PS. In comparison, the PS tries to achieve an accurate fitting only on given experimental data, but it may over- or under- estimate modulus values outside of the experimental range, resulting in likely false ${E}_{0}$ or ${E}_{\infty }$ values. The comparison between the new model and the PS (or GM model) is fair as we have used the same optimization scheme to determine/fit model parameters. When using the same amount of model parameters, the proposed model has achieved more unique solution than the PS as illustrated in the Supplementary A. Given a relatively very large term number of the GM model ($n\ge \,30$), its accuracy may be improved for the data fitting (very likely) and data prediction outside of experimental range (less likely). Some researchers used a pre-smooth method to improve the fitting accuracy of the PS^29^. However, the new model could be superior than the GM model or PS considering multiple aspects as follows: (1) a model like the new one with less model parameters that satisfies the fitting accuracy is often preferred for the simplicity purpose; (2) a large number of model parameters of the PS (GM model) can produce more variability with non-unique solutions for determining the model parameters using mathematical optimization schemes; (3) it is more difficult to explain the physical mechanism for the model with a relatively much larger number of model parameters other than improving the accuracy for fitting the experimental data; (4) the proposed model offers more flexibility, e.g. it is a standard solid-model when $\alpha =1$, and (5) we also include the nonlinear strain-hardening behavior in the new model.

Our numerical solution have also indicated that the new model has improved the prediction accuracy. It is stable numerically. It does not reduce computation speed for convergence as compared to the PS.

Therefore, the new model may be used as an alternative for describing viscoelastic behavior of a broad range of solid materials to improve the data prediction accuracy. However, as GM model does the model is unable to simulate the special or abnormal physical behaviors such as the stress overshot of some materials including structural glasses^50^ and amorphous solids^51^.

## Materials and Methods

We validated the model based on laboratory testing data and provided physical interpretation. We developed numerical solution – a FE method to implement the model for simulating responses of a number of different materials.

We conducted relaxation and frequency-sweep (dynamic modulus) tests for part of these materials to determine their relaxation and complex modulus, while the left experimental data were obtained from existing literature. For the dynamic modulus test on AC material, four temperatures and six frequencies were used. The spider silk has a very small size (i.e. 20 *μ*m)^48^. To measure the stress *vs*. strain behavior of the silk^48^, the material was adhered on a glass substrate under a unidirectional stretch (see Fig. 6(a)).

To fit model parameters from experimental data, we used the generalized reduced-gradient optimization algorithm which is one of the most frequently used optimization methods in practice. We also evaluated the model for predicting modulus values outside of the frequency or reduced-time range of experimental data, which was not (often) conducted by existing literature. By using the major middle part of the experimental data in time or frequency domain, we fitted the model parameters. Consequently, we used the fitted model to predict the modulus values outside of the time/frequency domain used for model fitting, and compared prediction values with the actual experimental measurements.

We developed a Galerkin-based FE method to implement the proposed $E(t)$ formula and derived its mathematical solution. The method is based on a time-domain scheme that integrates $E(t)\,$with time and space for a robust and fast computation^52–54^.

## Supplementary information

Supplementary information.
