# Supplementary material for: A new nonlinear viscoelastic model and mathematical solution of solids for improving prediction accuracy
Source: Sci Rep. 2020 Feb 10;10:2202. doi: 10.1038/s41598-020-58240-y (PMC7010732; doi:10.1038/s41598-020-58240-y)
Supplement: Supplementary file 1 — Supplementary information. [file 41598_2020_58240_MOESM1_ESM.docx]

A new nonlinear viscoelastic model and mathematical solution of solids for improving prediction accuracy

Qinwu Xu^1,2^, Björn Engquist1^2,3,^*, Mansour Solaimanian^4^, Kezhen Yan^1,^*

Supplementary Material

# Senstivity analysis of model parameters

We applied the proposed model to agar and bone materials for sensitivity analysis of model parameters. Model results show that a higher $\mu$ value results in higher $E(t)$ value without changing other model parameters (see Fig. 1a for the agar material); while a higher $\alpha$ value induces a higher modulus variation rate such that $\partial E(t)/\partial t\propto\alpha$ (see Fig. 1b for the bone material).

^1^School of Civil Engineering, Yango University, Fuzhou, Fujian 350015 China. ^2^Institute for Computational Engineering and Sciences and ^3^Department of Mathematics, the University of Texas at Austin, Austin, TX 78712 USA. ^4^Civil and Environmental Engineering, Penn State, State College, PA 16801 USA. Correspondence and requests for materials should be addressed to K. Yan (email:yankz2004@163.com) and B. Engquist (email: engquist@oden.utexas.edu).

**Supplementary Fig. 1. Model parameter sensitivity analysis for: (a) agar material (experimental data was reproduced from [41]. and (b) bone materials using proposed model (experimental data was reproduced from [1]). Note:** $\boldsymbol{\tau=1}\boldsymbol{s}$ **for constant temperature.**

# Model derivation for complex modulus

According the model format shown in Fig. 2, the stress and strain equilibrium satisfies the following (all in frequency domain):

$\varepsilon\left\{ e^{i\omega} \right\}=\varepsilon_{0}\left\{ e^{i\omega} \right\}$ (1)

$\varepsilon_{0}\left\{ e^{i\omega} \right\}=\varepsilon_{1}\left\{ e^{i\omega} \right\}+\varepsilon_{2}\left\{ e^{i\omega} \right\}$ (2)

$\sigma\left\{ e^{i\omega} \right\}=E_{\infty}\varepsilon_{0}\left\{ e^{i\omega} \right\}+E_{R}\varepsilon_{1}\left\{ e^{i\omega} \right\}$ (3)

$\sigma_{1}\left\{ e^{i\omega} \right\}=E_{R}\varepsilon_{1}\left\{ e^{i\omega} \right\}=\sigma_{2}\left\{ e^{i\omega} \right\}=$

${e^{+i\phi}\frac{\mu}{\tau}(\omega\tau)}^{1-\alpha}\varepsilon_{2}\left\{ e^{i\omega} \right\}$ (4)

$\varepsilon_{1}\left\{ e^{i\omega} \right\}+\varepsilon_{2}\left\{ e^{i\omega} \right\}=\varepsilon_{0}\left\{ e^{i\omega} \right\}$ (5)

$\eta$,$\mu,\tau$, $\sigma_{\eta}$, $\varepsilon_{2}$

$E0$*-*$E\infty$, $\sigma_{1}$, $\varepsilon_{1}$

$E_{\infty}$, $\sigma_{0}$, $\varepsilon_{0}$

$$\sigma\left\{ e^{i\omega} \right\},\varepsilon\left\{ e^{i\omega} \right\}$$

$$\sigma\left\{ e^{i\omega} \right\}$$

**Supplementary Fig. 2. Model format in spring-dashpot system.**

From Equation 4, the strain of $\varepsilon_{2}\left\{ e^{i\omega} \right\}$ can be derived as:

$\varepsilon_{2}\left\{ e^{i\omega} \right\}={E_{R}e^{-i\phi}\frac{\tau}{\mu}(\omega\tau)}^{\alpha-1}\varepsilon_{1}\left\{ e^{i\omega} \right\}$ (6)

Substitute Equation 6 into 5 to attain the ratio of ${\varepsilon_{1}/\varepsilon}_{0}$:

$\frac{\varepsilon_{1}\left\{ e^{i\omega} \right\}}{\varepsilon_{0}\left\{ e^{i\omega} \right\}}=\frac{1}{1+{{E_{R}e}^{-i\phi}\frac{\tau}{\mu}(\omega\tau)}^{\alpha-1}}$ (7)

Substitute Equation 7 to 3 and divide it by $\varepsilon\left\{ e^{i\omega} \right\}$ to attain the model expression for complex modulus as follows:

$E^{*}\left( \omega\right)=\frac{\sigma\left\{ e^{i\omega} \right\}}{\varepsilon\left\{ e^{i\omega} \right\}}=\frac{\sigma\left\{ e^{i\omega} \right\}}{\varepsilon_{0}\left\{ e^{i\omega} \right\}}=E_{\infty}+\frac{E_{R}}{{{E_{R}e}^{-i\phi}\frac{\tau}{\mu}(\omega\tau)}^{\alpha-1}+1}=E_{\infty}+\frac{E_{R}{\mu/\tau e}^{i\phi}{(\omega\tau)}^{1-\alpha}}{E_{R}+{\mu/\tau e}^{i\phi}{(\omega\tau)}^{1-\alpha}}$ (8)

# Thermodynamic consistency

To ensure thermodynamic consistency, the Calusius-Duhem inequality shall be satisfied [2] as follows:

$\rho cT=-\frac{\rho\partial\Pi}{\partial t}+\frac{\sigma\partial\varepsilon}{\partial t}-\rho s\frac{\partial T}{\partial t}+c\left( \frac{\partial T}{\partial x} \right)^{2}\geq0$ (9)

where $\rho Tc$ is a specific energy dissipation term, $c$ is thermal conductivity, $\Pi$ is the specific free energy, $\varepsilon$ is the total strain of the model system, and $s$ is the specific entropy.

The possibility of this inequality can be proven in the following.

The total strain can be decomposed into two parts as follows:

$\varepsilon=\varepsilon_{0}+\varepsilon_{T}=\left( \varepsilon_{1}+\varepsilon_{2} \right)+\varepsilon_{T}=\left( \varepsilon_{1}+\varepsilon_{2} \right)+\alpha_{T}\Delta T$ (10)

where $\varepsilon_{0}$ is the total mechanical strain of the model - the same as the strain posed by $E_{\infty}$, $\varepsilon_{T}$ is the thermal strain equals to $\alpha_{T}\Delta T$, $\alpha_{T}$ is the coefficient of thermal expansion, and $\Delta T$ is temperature variation of the model system.

The total stress can be decomposed into two parts:

$\sigma=\sigma_{0}+\sigma_{1}=\sigma_{0}+\sigma_{\eta}$ (11)

where $\sigma_{0}$ is the elastic stress at infinite time posed by $E_{\infty}$, and $\sigma_{\eta}$ is the stress posed by the dashpot – the same as that posed by spring network $E_{R}=E_{0}-E_{\infty}$.

The free energy of the model system can be given as:

$\Pi=\Pi_{0}\left( \varepsilon_{0},T \right)+\Pi_{1}\left( \varepsilon_{1},T \right)+\pi\left( T \right)$ (12)

$\Pi_{0}\left( \varepsilon_{0},T \right)$ is the energy stored by $E_{\infty}$, $\Pi_{1}\left( \varepsilon_{e},T \right)$ is the energy stored by the elastic network $E_{R}$, and $\pi(T)$ is a heat capacity related energy.

Substitute Equation 10 and Equation 12 into Equation 9 to reach the following equilibrium:

$\rho cT=-\rho\left( \frac{\partial\Pi_{0}}{\partial t}\left( \varepsilon_{0},T \right)+\frac{\partial\Pi_{1}}{\partial t}\left( \varepsilon_{1},T \right)+\frac{\partial\pi}{\partial t}\left( T \right) \right)+\sigma\left( \frac{\partial\varepsilon_{0}}{\partial t}+\frac{\partial\varepsilon_{T}}{\partial t} \right)-\rho s\frac{\partial T}{\partial t}+c\left( \frac{\partial T}{\partial x} \right)^{2}$ (13)

Substitute Equation 9 to 11 into Equation 13, and apply the chain rule to achieve the following:

$\rho cT=-\rho\left( \frac{{\partial\Pi}_{0}}{\partial\varepsilon_{0}}\frac{\partial\varepsilon_{0}}{\partial t}+\frac{{\partial\Pi}_{0}}{\partial T}\frac{\partial\varepsilon_{T}}{\partial t}+\frac{\partial\Pi_{1}}{\partial\varepsilon_{1}}\frac{\partial\varepsilon_{1}}{\partial t}+\frac{\partial\Pi_{1}}{\partial T}\frac{\partial\varepsilon_{T}}{\partial t}+\frac{\partial\pi}{\partial T}\frac{\partial T}{\partial t} \right)+\left[ \sigma_{0}\frac{\partial\varepsilon_{0}}{\partial t}+\sigma_{\eta}\left( \frac{\partial\varepsilon_{1}}{\partial t}+\frac{\partial\varepsilon_{2}}{\partial t} \right)+\sigma\alpha_{T}\frac{\partial T}{\partial t} \right]-\rho s\frac{\partial T}{\partial t}+c\left( \frac{\partial T}{\partial x} \right)^{2}$ (14)

This can ber rearranged as follows:

$\rho cT=\left( \sigma_{0}-\rho\frac{{\partial\Pi}_{0}}{\partial\varepsilon_{0}} \right)\frac{\partial\varepsilon_{0}}{\partial t}+\left( \sigma_{\eta}-\rho\frac{{\partial\Pi}_{1}}{\partial\varepsilon_{1}} \right)\frac{\partial\varepsilon_{1}}{\partial t}$

$+\left( {\sigma\alpha}_{T}+\frac{{\partial\Pi}_{0}}{\partial T}+\frac{\partial\pi}{\partial T}-\rho s-\rho\frac{{\partial\Pi}_{1}}{\partial T} \right)\frac{\partial T}{\partial t}+\sigma_{\eta}\frac{\partial\varepsilon_{2}}{\partial t}+c\left( \frac{\partial T}{\partial x} \right)^{2}$ (15)

$\frac{\partial\varepsilon_{0}}{\partial t},\frac{\partial\varepsilon_{1}}{\partial t},\frac{\partial T}{\partial t}$ can be arbitrary, and thus the coefficients of the terms of $\frac{\partial\varepsilon_{0}}{\partial t},\frac{\partial\varepsilon_{1}}{\partial t},\frac{\partial T}{\partial t}$ have to vanish to satisfy $\rho T\gamma\geq0$. Therefore, Equation 15 may be reduced to:

$\rho cT=\sigma_{\eta}\frac{\partial\varepsilon_{2}}{\partial t}+c\left( \frac{\partial T}{\partial x} \right)^{2}$ (16)

The stress of the viscous dashpot can be expressed as $\sigma_{\eta}=\eta\frac{\partial\varepsilon_{2}}{\partial t}$ ($\eta$ is viscosity of the dashpot) and thus Equation 16 satisfies:

$\rho cT=\eta\left( \frac{\partial\varepsilon_{2}}{\partial t} \right)^{2}+\gamma\left( \frac{\partial T}{\partial x} \right)^{2}\geq0$ (17)

# Experimental validation

The model parameters are determined by fitting on experimental data using optimization skills. The nonlinear reduced gradient method was used to fit the model parameters by minimizing the objective function $f\left( x_{1},x_{2},\ldots.,x_{m} \right) \forall x_{i}>0$:

$f\left( x_{1},x_{2},\ldots.,x_{m} \right) ={\sum_{i}^{N} \left[ E_{i}\left( x_{1},x_{2},\ldots.,x_{m} \right)-\hat{E}_{i} \right]}^{2}$ (18)

where $x_{i}$ is the model parameters, e.g., for generalized Maxwell (GM) model $x_{i}=E_{i}$ and $\eta_{i}$, $N$ is the total number of experimental data points, $E_{i}$ and $\hat{E_{i}}$ is the $i^{th}$ modeled and measured modulus value, respectively.

For this optimization method, the gradients (derivatives with respect to each model parameter) are calculated based on the central finite difference (CFD) method. The iteration goal is to satisfy the first order essential optimal condition (i.e. the gradient is or close to zero).

It is known that inverse computation is generally dependent on seed values and it may turn out multiple results of model parameters which all satisify the optimization objective. This is especially true for the model with higher number of parameters such as GM model and its Prony series (PS) formula. Fig. 3 illustrates the optimization results for the PS with $n=2$ (standard solid model) and the proposed model (both have five model parameters) using different seed values that consdier three general cases: 1) modulus valus are higher, 2) lower, and 3) close to as compared to true values. The seeds and output values of model parametr are listed in Table 1**.** Results have shown that the proposed model yilds more unique solutions and $E(t)$ shapes than the PS when using different seed values, indicating its higher stability (see Fig. 3a *vs.* 3b). When using a relatively large term number for the PS, its variability (e.g. different combinations of $E_{i}$ and $\eta_{i}$ values) can be larger, and it could be difficult to estimate property seed values for achiving more unique solutions. In comparison, the porposefd model uses much leass model parameters , but it is still able to caputure the full time range. It is also easier to estimate proper seed values such as $E_{\infty}$ and $E_{0}$ values according to the expeimrental data range.

**Supplementary Fig. 3. Optimization analysis: (a) Prony series with** $\boldsymbol{n}$**=2 with 5 model parameters and (b) proposed model for polyurethane polymer materials.**

**Supplementary Table 1. Model seed and output after optimization to fit experimental data.**

1. GM model ($n$=1) or standard solid model

1. Proposed model

Note: $\tau=1$ for constant temperature.

We present model fitting and prediction results of an agar material in Supplementary Fig. **4**. Result shows that the proposed model for agar material attained relatively close results for fitting and predictions (see Supplementary Fig. **4**a). In comparison, the GM model has slightly over-predicted $E(t)$ at high time range and produced less stability in the low time ranges (Supplementary Fig. **4**b).

**Supplementary Fig. 4. Model fit and prediction of agar material: (a) proposed model and (b) proposed model versus the GM model and Prony series (experimental data of agar was reproduced from [3]).**

We present model fitting and prediction results of a bovine bone material in Supplementary **Fig**. **5**. Result shows that the proposed model produces a smoother and still accurate curve fitting. The PS over-predicts $E(t)$ at the high time range and produced gaps to measurement values as it intends to rapidly converge to $E_{\infty}$ once it is outside of the time range of fitted data.

**Supplementary Fig. 5. Model fit and prediction of bovine femoral bones (experimental data of bone was reproduced from ([4]).**

Table 2 lists the fitted model parameters of the GM model for the agarose gel, vaginal tissue, bone and agar materials.

**Supplementary Table 2. Fitted model parameters of GM model (**$\boldsymbol{n}$**=14): a). agarose gel; b) vaginal tissue; c) bone; and d) agar materials.**

1. b)
2. d)
3. N. Sasaki and M. Yoshikawa (1993) Stress relaxation in native and EDTA-treated bone as a function of mineral content. Journal of Biomechanics **26**, 77-83.
4. Haupt P. 1993 Non-equilibrium thermodynamics with applications to solids. In *CISM-Course 336* (ed. W. Muschik), pp. 138–140. New York, NY: Springer.
5. Shen J, Cheng P, Gu W, Hao Z. 2013 Stress relaxation measurement of viscoelastic materials using a polymer-based microfluidic device. *Sens. Actuators, A* **203**, 119–130. (doi:10.1016/j.sna.2013.08.038)
6. Sasaki N. 2012 Viscoelastic properties of biological materials. In *Viscoelasticity - From theory to biological applications* (ed. JD Vincente), pp. 99–122. Intech.
